# Longitudinal measurement invariance and explanatory IRT models for adolescents’ oral health-related quality of life

**DOI:** 10.1186/s12955-018-0879-x

**Published:** 2018-04-11

**Authors:** David T. W. Yau, May C. M. Wong, K. F. Lam, Colman McGrath

**Affiliations:** 1 Dental Public Health, Faculty of Dentistry, The University of Hong Kong, Pokfulam, Hong Kong; 2 Department of Statistics and Actuarial Science, Faculty of Science, The University of Hong Kong, Pokfulam, Hong Kong

**Keywords:** Oral health-related quality of life, Longitudinal invariance, Item response theory, Child perception questionnaires, Graded response model

## Abstract

**Background:**

Longitudinal invariance is a perquisite for a valid comparison of oral health-related quality of life (OHRQoL) scores over time. Item response theory (IRT) models can assess measurement invariance and allow better estimation of the associations between predictors and latent construct. By extending IRT models, this study aimed to investigate the longitudinal invariance of the two 8-item short forms of the Child Perception Questionnaire (CPQ_11–14_) regression short form (RSF:8) and item-impact short form (ISF:8) and identify factors associated with adolescents’ OHRQoL and its change.

**Methods:**

All students from S1 and S2 (equivalent to US grades 6 and 7) who were born in April 1997 and May 1997 (at age 12) from 45 randomly selected secondary schools were invited to participate in this study and followed up after 3 years. Data on the CPQ_11–14_ RSF:8 and CPQ_11–14_ ISF:8, demographics, oral health behavior and status were collected. Explanatory graded response models were fitted to both short forms of the CPQ_11–14_ data for assessing longitudinal invariance and factors associated with OHRQoL. The Bayesian estimation method – Monte Carlo Markov Chain (MCMC) with Gibbs sampling was adopted for parameter estimation and the credible intervals were used for inference.

**Results:**

Data from 649 children at age 12 at baseline and 415 children at age 15 at follow up were analyzed. For the 12 years old children, healthier oral health behavior, better gum status, families with both parents employed and parents’ education level were found to be associated with better OHRQoL. Four items among the 2 short forms lacked longitudinal invariance. With statistical adjustment of longitudinal invariance, OHRQoL were found improved in general over the 3 years but no predictor was associated with OHRQoL in follow-up. For those with decreased family income, their OHRQoL had worsened over 3 years.

**Conclusions:**

IRT explanatory analysis enables a more valid identification of the factors associated with OHRQoL and its changes over time. It provides important information to oral healthcare researchers and policymakers.

**Electronic supplementary material:**

The online version of this article (10.1186/s12955-018-0879-x) contains supplementary material, which is available to authorized users.

## Background

There has been a growing concern of measuring an individual’s oral health-related quality of life (OHRQoL) in health research in the past two decades. OHRQoL is an abstract concept which cannot be directly observed and different measurement instruments have been developed over the years. It is an essential prerequisite to assess the psychometric properties of an instrument before it can be adopted and use appropriately in research.

In studying OHRQoL, rating scales on frequency or severity of conditions described by items are often used and a number is assigned to each response option, for example, “Never” = 0, “Once/ twice” = 1 and “Sometimes” = 2 …,etc. The total score is typically used as the participant’s score of OHRQoL. This scoring method assumes that each item equally relates to the overall OHRQoL level and the response options are in interval scale, which may not be complied in reality. Alternatively, item response theory (IRT) can be used to analyze individuals’ OHRQoL without the above assumptions [1]. IRT models relates the probability of answering certain options of each item in relation to (1) the respondents’ level of latent construct (i.e. OHRQoL) and (2) the measurement properties (reflected by discriminatory and threshold parameters) of each item. IRT has been commonly applied to evaluate the measurement properties of measurement including OHRQoL instruments [2, 3].

Child Perceptions Questionnaire (CPQ_11–14_) regression short form (RSF:8) and item-impact short form (ISF:8) are 2 versions of 8-item questionnaire commonly used in measuring OHRQoL for children aged at around the onset of puberty [4]. The 8-item forms CPQ_11–14_ (RSF:8 and ISF:8) have been extensively validated in Hong Kong using not only conventional approaches, but also factor analysis and IRT [2, 5, 6]. Children at this stage are characterized by increasing centrality of peer crown and their pre-occupation with others’ view of self [7]. With this rapid psychosocial development, their perception of CPQ_11–14_ items may change as they grow up. For example, a rating of “2” in the follow-up questionnaire may be interpreted in the same way as a rating of “1” at baseline [8]. Therefore, statistical adjustment is needed to ensure the CPQ_11–14_ questionnaire administered at different ages are comparable (i.e. the concept of longitudinal invariance). The establishment of longitudinal invariance help warrant that any change in the score is attributed only to the true change in OHRQoL instead of a change in conceptualization, interpretation or recalibration of the measurement scale over time [9].

Besides the assessment of the psychometric properties of an instrument, researchers are often interested in investigating factors explaining the OHRQoL using explanatory analysis. It is commonly performed with the sum score of the OHRQoL items as the outcome measurement in multiple linear regressions. However, this approach again ignores the ordinal nature of the response options [1]. Some researchers have made use of the latent score estimated form IRT model (step 1) and subsequently use the score to perform regression analysis (step 2). However, this 2-step approach may yield biased standard error of the regression coefficients due to the possible violation of homoscedasticity assumption of linear regression [10]. Extending the IRT model by including predictors (explanatory item response analysis) helps (1) quantify the heteroskedasticity and (2) secure a more reliable inference of parameter by simultaneously estimating the item parameters, latent OHRQoL scores and regression coefficients [11, 12] .

Explanatory IRT analysis allows adjustment for any longitudinal invariance and provided a more reliable inference on regression coefficients [13]. This study aimed to explore factors contributing to OHRQoL of adolescents at age 12 and 15, and its change with statistical adjustment for longitudinal invariance under the framework of IRT. This article endeavors to achieve 3 objectives: (1) investigating factors associated with OHRQoL using the 2 short forms of CPQ_11–14_ (RSF:8 and ISF:8) at 12 years old by an explanatory item response model; (2) investigating the longitudinal invariance of the 2 short forms under the framework of IRT; and (3) assessing the effect of demographics, behavioral and clinical factors on OHRQoL of adolescents aged 12 and 15, and its change using three explanatory item response models while adjusting for any longitudinal invariance.

## Methods

### Sample

The participants in this study were secondary school students recruited for an observation survey to study the association between dental caries and adiposity status [14]. Baseline data were collected from January to April 2010 and they were followed up after 3 years in January to April 2013. The primary sampling unit was Hong Kong secondary schools and the sampling frame was the list of Hong Kong local secondary schools. 45 local secondary schools (about 10% of local secondary school) were randomly drawn. Within each secondary school, all students from S1 and S2 (equivalent to US grades 6 and 7) who were born in April 1997 and May 1997 were invited to participate in this study. The participants were ensured to be 12 and 15 years old respectively at baseline and follow-up. The study protocol was approved by Institutional Review Board of the University of Hong Kong/ Hospital Authority Hong Kong West Cluster (WU09–435) and written parental consent was obtained.

### Measure

Participants were asked to complete a questionnaire in Chinese, consisted of both CPQ_11–14_ RSF:8 and ISF:8, and questions concerning their global self-health ratings, demographic (place of birth and years of residency in Hong Kong) and oral health behaviors (snacking frequency between meals, brushing frequencies, use of fluoridated toothpaste, and previous participation of the School Dental Care Service (SDCS)). Parents were asked about the families’ social economic status (SES) (parents’ place of birth, employment status, family income, education level and whether they have lived in Hong Kong for 7 years or more). Participants and their parents completed the questionnaires in a self-administered manner.

In each item of CPQ_11–14_, participants were asked “In the past 3 months, how often have you … (had/been)…because of your teeth/mouth?”. The five-point scale were: “Never” = 0; “Once/ twice” = 1; “Sometimes” = 2; “Often” = 3; “Every day/ almost every day”=4 [15]. The possible total score ranges of both CPQ_11–14_ RSF:8 and ISF:8 is 0–32 (with a lower score indicates better OHRQoL). “Never” = 0 were imputed for the missing responses at baseline and follow-up (1.4% of all responses) because it was presumed that children not answering the items probably have not encountered the situation described. This imputing method was previously used to handle another OHRQoL questionnaire with a “Don’t know” option [3]. Respondents with more than 2 missing items were excluded in order to avoid over imputation.

Anchor items were used to equate the baseline (at age 12) and follow-up (at age 15) questionnaires. Since no prior information on invariant items of CPQ_11–14_ have been available, five questions concerning global self-health rating were asked, namely “How do you rate your dental/ oral health condition?” (“Very Good” /“Good” /“Average” /“Bad” /“Very Bad”), “How does your dental/oral health impact your life?” (“Not at all” /“A little” /“Somewhat” /“Greatly” /“Tremendously”), “To what extent have you been bothered by these problems?” (i.e. the problems described in the 8 item short form CPQ_11–14_), “To what extent have these problems affected your quality of life?” and “To what extent have these problems affected your life overall?” (“Never” /“Hardly Ever” /“Occasionally” /“Often” /“Very Often”).

### Clinical examination

Each participant received an oral examination for assessment of dental caries experience and periodontal condition following the World Health Organization’s recommendation [16]. Dental caries experiences were measured by the number of decayed, missing and filled teeth (DMFT). Periodontal conditions were measured by the community periodontal index (CPI) with 3 levels, namely “Healthy gum/ absence of bleeding or calculus”=0, “Bleeding on probing”=1 and “Calculus deposits” = 2 on 6 index teeth (16, 11, 26, 36, 31 and 46). The highest CPI score was used as a summary measure of periodontal health status. All oral examinations were performed by trained and calibrated dentists in the school premises on a portable dental chair. The oral examinations were performed using intra-oral disposable mouth mirrors with LED light and CPI probes by one examiner at baseline and another one in the follow-up study. About 10% of the participants were re-examined to evaluate the intra examiner reliability using Intra-class correlation (ICC) and Kappa Statistics, which indicated the consistency in measuring DMFT and CPI respectively (DMFT: ICC = 0.99 at age 12, ICC = 0.82 at age 15; CPI: Kappa = 0.74 at age 12, Kappa = 0.84 at age 15). Anthropometric assessment was also conducted but the information was not used in this study.

### Statistical models

#### Explanatory GRM

The graded response model (GRM) was extended by incorporating variables (listed in Tables 1 and 2) to explain the latent construct – OHRQoL. The explanatory GRM with p explanatory variables, J items and K response options is formulated as

$$ \log \left(\frac{{P^{+}}_{jk}}{1-{P^{+}}_{jk}}\right)={\mathrm{a}}_{\mathrm{j}}\kern0.1em \left(\theta -{\mathrm{b}}_{\mathrm{j}\mathrm{k}}\right), $$

$$ \mathrm{where}\ \theta ={\mathrm{x}}_1{\upgamma}_1+{\mathrm{x}}_2{\upgamma}_2+\dots +{\mathrm{x}}_{\mathrm{p}}{\upgamma}_{\mathrm{p}}+\upvarepsilon; $$

**Table 1:** The frequency distribution of background variables

Factors		Baseline (*n* = 649)		Followed- up (*n* = 415)	
		*n*	%	*n*	%
Gender^a^	Boys	319	49.2	–	**–**
	Girls	330	50.8	–	**–**
Father’s education level^a^	College graduate or above	81	12.5	–	**–**
	Senior secondary school (S4‑7)	313	48.2	–	**–**
	Junior Secondary school (S1‑3)	167	25.7	–	**–**
Mother’s education level^a^	Below Secondary school	88	13.6	–	**–**
	College graduate or above	110	16.9	–	**–**
	Senior secondary school (S4‑7)	267	41.1	–	**–**
	Junior Secondary school (S1‑3)	184	28.4	–	**–**
	Below Secondary school	88	13.6	–	**–**
Student Dental Care Service/ Dental insurance^a^	Yes	636	98.0	–	**–**
	No	13	2.0	–	**–**
Monthly family income	>$40,000	96	14.8	81	19.5
	$30,000‑$40,000	65	10.0	42	10.1
	$20,000‑$30,000	101	15.6	69	16.6
	$10,000‑$20,000	226	34.8	165	39.8
	<$10,000	161	24.8	58	14.0
Snacking frequency between meals	3 times or more	54	8.3	38	9.2
	Twice	123	19.0	81	19.5
	Once	334	51.4	202	48.7
	None	138	21.3	94	22.7
Daily brushing frequency	Twice or more	467	72.0	303	73.0
	Less than twice	182	28.0	112	27.0
Both parents employed	Yes	417	64.3	276	66.5
	No	232	35.7	139	33.5
Maximum CPI	Healthy gum/ No bleeding or calculus	89	13.7	44	10.6
	Bleeding on probing	139	21.4	46	11.1
	Calculus deposits	421	64.9	325	78.3
DMFT	0	446	68.7	193	46.5
	1	109	16.8	67	16.1
	2	52	8.0	47	11.3
	> 3	42	6.5	108	26.1
	Mean (s.d.)	0.55	(1.01)	1.70	(2.34)

^a^Only collected in the baseline study

**Table 2:** Regression coefficients with 95% credible intervals of explanatory GRM

Factors	Comparison of regression coefficients	CPQ_11‑14_ RSF:8	CPQ_11‑14_ ISF:8
Gender	Boys vs. Girls	‑0.12 (‑0.30,0.07)	‑0.01 (‑0.21,0.19)
Both parents employed	Yes vs. No	**‑0.20 (‑0.40,0.00)**	**‑0.28 (‑0.51,‑0.07)**
Monthly family income	>$40,000 vs. <$10,000	0.00 (‑0.38,0.37)	0.23 (‑0.18,0.63)
	$30,000‑$40,000 vs. <$10,000	0.30 (‑0.06,0.68)	0.34 (‑0.05,0.75)
	$20,000‑$30,000 vs. <$10,000	0.12 (‑0.20,0.44)	0.15 (‑0.19,0.49)
	$10,000‑$20,000 vs. <$10,000	0.12 (‑0.12,0.36)	0.17 (‑0.11,0.44)
	>$40,000 vs. $30,000‑$40,000	‑0.30 (‑0.68,0.07)	‑0.11 (‑0.51,0.28)
	>$40,000 vs. $20,000‑$30,000	‑0.11 (‑0.48,0.24)	0.08 (‑0.32,0.46)
	>$40,000 vs. $10,000‑$20,000	‑0.11 (‑0.45,0.22)	0.06 (‑0.31,0.42)
	$30,000‑$40,000 vs. $20,000‑$30,000	0.19 (‑0.17,0.55)	0.19 (‑0.22,0.58)
	$30,000‑$40,000 vs. $10,000‑$20,000	0.19 (‑0.15,0.52)	0.18 (‑0.20,0.53)
	$20,000‑$30,000 vs. $10,000‑$20,000	0.00 (‑0.27,0.27)	‑0.01 (‑0.32,0.29)
Father’s education	College graduate or above vs. Below secondary school	‑0.35 (‑0.82,0.05)	‑0.45 (‑0.93,0.00)
	Senior secondary school (S4‑7) vs. Below secondary school	**‑0.49 (‑0.80,‑0.21)**	**‑0.49 (‑0.83,‑0.15)**
	Junior Secondary school (S1‑3) vs. Below secondary school	**‑0.59 (‑0.91,‑0.30)**	**‑0.51 (‑0.84,‑0.17)**
	College graduate or above vs. Senior secondary school (S4‑7)	0.13 (‑0.20,0.46)	0.03 (‑0.33,0.39)
	College graduate or above vs. Junior secondary school (S1‑3)	0.24 (‑0.16,0.64)	0.06 (‑0.39,0.47)
	Senior secondary school (S4‑7) vs. Junior secondary school (S1‑3)	0.11 (‑0.15,0.35)	0.02 (‑0.25,0.29)
Mother’s education	College graduate or above vs. Below secondary school	0.29 (‑0.11,0.70)	0.33 (‑0.11,0.77)
	Senior secondary school (S4‑7) vs. Below secondary school	0.26 (‑0.05,0.58)	**0.39 (0.04,0.73)**
	Junior Secondary school (S1‑3) vs. Below secondary school	**0.50 (0.18,0.81)**	**0.48 (0.14,0.80)**
	College graduate or above vs. Senior secondary school (S4‑7)	0.03 (‑0.27,0.33)	‑0.06 (‑0.40,0.27)
	College graduate or above vs. Junior secondary school (S1‑3)	‑0.21 (‑0.56,0.13)	‑0.15 (‑0.53,0.23)
	Senior secondary school (S4‑7) vs. Junior secondary school (S1‑3)	‑0.24 (‑0.49,0.00)	‑0.09 (‑0.35,0.17)
Brushing frequency	Twice or more daily vs. Less than twice daily	**‑0.21 (‑0.40,0.00)**	**‑0.33 (‑0.55,‑0.10)**
Snacking frequency	3 times or more vs. None	**0.47 (0.12,0.82)**	**0.47 (0.07,0.86)**
between meals	Twice vs. None	0.25 (‑0.02,0.54)	0.24 (‑0.07,0.57)
	Once vs. None	‑0.05 (‑0.30,0.18)	‑0.03 (‑0.29,0.24)
	3 times or more vs. Twice	0.21 (‑0.14,0.57)	0.22 (‑0.17,0.62)
	3 times or more vs. Once	**0.52 (0.18,0.86)**	**0.50 (0.15,0.85)**
	Twice vs. Once	**0.31 (0.07,0.54)**	**0.28 (0.01,0.53)**
CPI	Calculus deposits vs. Healthy gum /No bleeding or calculus	0.20 (‑0.05,0.47)	**0.39 (0.08,0.73)**
	Bleeding on probing vs. Healthy gum /No bleeding or calculus	0.26 (‑0.04,0.57)	**0.40 (0.05,0.76)**
	Calculus deposits vs. Bleeding on probing	‑0.06 (‑0.28,0.17)	‑0.02 (‑0.26,0.24)
DMFT		0.02 (‑0.07,0.11)	‑0.01 (‑0.11,0.09)

Data in boldface: 95% credible interval excluded 0, considered as statistically significant at 0.05 level of significance

a_j_ = item discriminatory parameter for the j^th^ item; b_jk_ = item threshold parameter for the k^th^ response option in the j^th^ item; j = 1,2,3…J; k = 1,2,3…K-1; P^+^_jk_ is the probability of choosing the k + 1^th^ or higher response options in the j^th^ item. The latent score (θ) consists of a linear combination of explanatory variables and regression coefficients (x_1_γ_1_ + x_2_γ_2_ + … + x_p_γ_p_), plus an error term (ε) [17]. 14 items were fitted into the GRM model (8 items in RSF:8 *plus* 8 items in ISF:8 *minus* 2 overlapping items among the 2 short forms).

The explanatory GRM was used to investigate the baseline factors associated with OHRQoL at 12 years old. The explanatory variables included demographic variables (place of birth and year of residency in Hong Kong), oral health behaviors (snacking frequency between meals, brushing frequencies, use of fluoridated toothpaste, and previous participation of the School Dental Care Service (SDCS)), the family social economic status (parents’ place of birth, employment status, family income, education level and whether they have lived in Hong Kong for 7 years or more), DMFT and CPI. Each categorical variable was recoded as dummy variables and included into the models.

The Bayesian estimation method – Monte Carlo Markov Chain (MCMC) with Gibbs sampling was adopted for parameter estimation and implemented via WinBUGS (Additional file 1) [18]. Non-informative priors were used and the posterior distribution was constructed using 12,000 simulations after 8000 burn-ins. The parameter estimates resulted from the use of non-informative priors can resemble the maximum likelihood estimation in the classical (frequentist) approach [19]. The 95% credible intervals of the parameters (95% probability that the true parameters is within the interval) were obtained from the 12,000 simulations. Associations of explanatory variables with OHRQoL were established when the 95% credible interval excluded 0 or difference between each pair of regression coefficients within a factor excluded 0.

#### GRM for assessing longitudinal invariance with external anchor items

Longitudinal invariance of the questionnaire (Table 3) was investigated by the GRM with varying discriminatory (a_jt_) and threshold parameters (b_jkt_) across age 12 and 15 years old. The model is formulated as:

$$ \ln \left(\frac{{P^{+}}_{jkt}}{1-{P^{+}}_{jkt}}\right)={\mathrm{a}}_{\mathrm{jt}}\left({\theta}_{\mathrm{t}}-{\mathrm{b}}_{\mathrm{jkt}}\right) $$

where *t* = 1 (at 12 years old) or 2 (at 15 years old). Meanwhile the scores (θ_t_) of the same respondents across the 2 time points were allowed to be correlated. Since too few respondents chose “Every day/ almost every day”=4 in some CPQ_11–14_ items, response options “Often” = 3 and “Every day/ almost every day” = 4 were combined in the longitudinal invariance analysis.

**Table 3:** GRM for assessing longitudinal invariance

	Median differences between baseline and follow up(credible intervals)			
	a	b_1_	b_2_	b_3_^a^
CPQ_11‑14_ RSF:8				
1. Mouth sores	‑0.16 (‑0.40,0.08)	‑1.65 (‑5.62,0.29)	‑0.58 (‑1.48,0.16)	2.75 (‑2.35,12.67)
2. Bad breath	0.01 (‑0.25,0.29)	‑0.74 (‑3.57,2.46)	‑0.15 (‑0.96,0.74)	‑0.05 (‑4.18,3.31)
3.Trouble sleeping	0.35 (‑0.02,0.70)	0.08 (‑0.24,0.42)	‑0.05 (‑0.85,0.63)	‑0.57 (‑2.23,0.77)
4. Difficult to say any words	0.01 (‑0.37,0.39)	‑0.34 (‑0.69,0.01)	‑0.41 (‑1.27,0.36)	‑1.34 (‑3.29,0.14)
5. Concerned with other people think	‑0.05 (‑0.38,0.30)	‑0.17 (‑0.45,0.12)	‑0.06 (‑0.40,0.28)	‑0.15 (‑0.99,0.66)
6. Upset	0.07 (‑0.33,0.47)	0.20 (‑0.04,0.44)	0.10 (‑0.31,0.51)	‑0.17 (‑1.18,0.82)
7. Argued with other children or your family	0.05 (‑0.35,0.44)	**0.48 (0.20,0.78)**	0.49 (‑0.02,1.04)	1.04 (‑0.38,2.77)
8. Teased/ called names by other children	0.15 (‑0.20,0.52)	**0.88 (0.52,1.29)**	**0.76 (0.05,1.57)**	0.89 (‑0.47,2.42)
CPQ_11‑14_ ISF:8				
1. Bad breath	0.01 (‑0.25,0.29)	‑0.74 (‑3.57,2.46)	‑0.15 (‑0.96,0.74)	‑0.05 (‑4.18,3.31)
2. Food caught between/ in teeth	‑0.1 (‑0.39,0.18)	‑1.87 (‑5.53,1.08)	‑1.13 (‑2.93,0.34)	‑0.75 (‑1.88,0.36)
3. Difficult to bite or chew food like apples, corn on the cob or steak	0.00 (‑0.36,0.36)	‑0.21 (‑0.48,0.06)	‑0.46 (‑1.08,0.11)	‑0.63 (‑1.91,0.50)
4. Difficult to drink or eat hot or cold food	‑0.18 (‑0.50,0.13)	**‑0.48 (‑0.83,‑0.13)**	0.11 (‑0.61,0.89)	1.01 (‑0.58,2.85)
5. Irritable/ frustrated	‑0.16 (‑0.53,0.18)	0.27 (‑0.01,0.56)	**0.53 (0.08,1.06)**	0.72 (‑0.32,1.91)
6. Upset	0.07 (‑0.33,0.47)	0.20 (‑0.04,0.44)	0.10 (‑0.31,0.51)	‑0.17 (‑1.18,0.82)
7. Avoided smiling/ laughing when around other children	0.39 (‑0.07,0.85)	‑0.23 (‑0.51,0.02)	‑0.26 (‑0.82,0.22)	‑0.3 (‑1.37,0.69)
8. Asked questions about your teeth, lips, jaws or mouth by others	0.07 (‑0.27,0.41)	‑0.09 (‑0.38,0.21)	‑0.43 (‑1.05,0.16)	‑0.85 (‑2.44,0.57)

^a^The options “Often” = 3 and “Every day/ almost every day” = 4 were combined

Data in boldface: 95% credible interval excluded 0, considered as statistically significant at 0.05 level of significance

In order to separate the change in OHRQoL arise from the interpretation of items, 5 external anchor items (global self-rating items of OHRQoL and their perceived impact on OHRQoL) which reflect their own perceptions of OHRQoL, were used to equate the questionnaires administered to respondents at both time points onto the same scale [20]. The discriminatory and threshold parameters of the anchor items were set equal at both time points (a_j1_ = a_j2_; b_jk1_ = b_jk2_).

Using WinBUGS, the differences in the parameters across the 2 time points and their corresponding credible intervals were computed. Significant parameter drift were inferred when the 95% credible interval of change in item parameters excluded 0. Items with significant parameter drift were considered as biased (lack of longitudinal invariance).

#### Longitudinal explanatory GRM

For modeling the change in OHRQoL over the 3 years, δ is defined as the difference in OHRQoL at 15 years old (θ_2_) and 12 years old (θ_1_), i.e. δ = θ_2_- θ_1_. Three models relating the explanatory variables to OHRQoL in a longitudinal context are adopted:

1. Presuming the change in OHRQoL is attributed to their status at baseline: baseline explanatory variables (listed in Table 4) were used to explain the change in OHRQoL, i.e. δ = x_11_γ_11_ + x_21_γ_21_ + … + x_p1_γ_p1_+ ε.
2. Presuming the change in OHRQoL is attributed to the change in status over 3 years: change in explanatory variables (listed in Table 5) were used to explain the change in OHRQoL, i.e. δ = x_1d_γ_1_ + x_2d_γ_2_ + … + x_pd_γ_p_ + ε, where x_pd_ represents the change in the explanatory variables x_p_ over the 3 years.
3. Presuming the effect of baseline and follow up factors on OHRQoL at their respective time point is of interest: baseline explanatory variables were used to explain the baseline OHRQoL; explanatory variables obtained in follow-up study were used to explain the follow-up OHRQoL (listed in Table 6). Static variables were used to explain both baseline and follow-up OHRQoL, i.e. θ_t_ = x_1t_γ_1t_ + x_2t_γ_2t_ + … + x_pt_γ_pt_ + ε_t,_ where *t* = 1, 2.

**Table 4:** Longitudinal explanatory GRM (Model 1): Regression coefficients with 95% credible intervals

Factors	Comparison of regression coefficients	CPQ_11‑14_ RSF:8	CPQ_11‑14_ ISF:8
Gender	Boys vs. Girls	‑0.26 (‑0.57,0.04)	‑0.20 (‑0.49,0.08)
Both parents employed	Yes vs. No	0.11 (‑0.22,0.42)	0.21 (‑0.11,0.51)
Monthly family income	>$40,000 vs. <$10,000	0.08 (‑0.54,0.72)	0.15 (‑0.45,0.71)
	$30,000‑$40,000 vs. <$10,000	0.08 (‑0.51,0.70)	0.01 (‑0.58,0.59)
	$20,000‑$30,000 vs. <$10,000	0.21 (‑0.31,0.69)	0.09 (‑0.36,0.54)
	$10,000‑$20,000 vs. <$10,000	0.04 (‑0.36,0.45)	‑0.14 (‑0.52,0.22)
	>$40,000 vs. $30,000‑$40,000	‑0.01 (‑0.61,0.62)	0.13 (‑0.46,0.72)
	>$40,000 vs. $20,000‑$30,000	‑0.12 (‑0.73,0.47)	0.05 (‑0.52,0.61)
	>$40,000 vs. $10,000‑$20,000	0.04 (‑0.55,0.61)	0.28 (‑0.25,0.82)
	$30,000‑$40,000 vs. $20,000‑$30,000	‑0.12 (‑0.72,0.48)	‑0.08 (‑0.67,0.48)
	$30,000‑$40,000 vs. $10,000‑$20,000	0.04 (‑0.51,0.59)	0.15 (‑0.4,0.68)
	$20,000‑$30,000 vs. $10,000‑$20,000	0.16 (‑0.29,0.59)	0.24 (‑0.18,0.65)
Father’s education	College graduate or above vs. Below secondary school	0.32 (‑0.47,1.04)	0.39 (‑0.26,1.09)
	Senior secondary school (S4‑7) vs. Below secondary school	0.19 (‑0.40,0.70)	0.17 (‑0.29,0.62)
	Junior Secondary school (S1‑3) vs. Below secondary school	0.29 (‑0.24,0.83)	0.29 (‑0.15,0.76)
	College graduate or above vs. Senior secondary school (S4‑7)	0.14 (‑0.39,0.69)	0.23 (‑0.30,0.76)
	College graduate or above vs. Junior secondary school (S1‑3)	0.02 (‑0.62,0.68)	0.10 (‑0.51,0.75)
	Senior secondary school (S4‑7) vs. Junior secondary school (S1‑3)	‑0.11 (‑0.52,0.30)	‑0.12 (‑0.52,0.26)
Mother’s education	College graduate or above vs. Below secondary school	‑0.40 (‑1.06,0.35)	‑0.57 (‑1.20,0.02)
	Senior secondary school (S4‑7) vs. Below secondary school	‑0.40 (‑0.88,0.16)	‑0.41 (‑0.87,0.06)
	Junior Secondary school (S1‑3) vs. Below secondary school	‑0.44 (‑0.95,0.08)	‑0.42 (‑0.87,0.02)
	College graduate or above vs. Senior secondary school (S4‑7)	‑0.01 (‑0.51,0.50)	‑0.17 (‑0.65,0.29)
	College graduate or above vs. Junior secondary school (S1‑3)	0.04 (‑0.53,0.63)	‑0.15 (‑0.69,0.37)
	Senior secondary school (S4‑7) vs. Junior secondary school (S1‑3)	0.05 (‑0.35,0.47)	0.02 (‑0.36,0.40)
Brushing frequency	Twice or more daily vs. Less than twice daily	‑0.19 (‑0.55,0.15)	‑0.12 (‑0.42,0.20)
Snacking frequency between meals	3 times or more vs. None	**‑1.12 (‑1.76,‑0.46)**	**‑0.91 (‑1.51,‑0.32)**
	Twice vs. None	‑0.21 (‑0.67,0.26)	‑0.01 (‑0.45,0.43)
	Once vs. None	‑0.01 (‑0.38,0.40)	0.21 (‑0.14,0.57)
	3 times or more vs. Twice	**‑0.91 (‑1.57,‑0.26)**	**‑0.9 (‑1.52,‑0.28)**
	3 times or more vs. Once	**‑1.11 (‑1.71,‑0.52)**	**‑1.13 (‑1.69,‑0.57)**
	Twice vs. Once	‑0.20 (‑0.60,0.19)	‑0.22 (‑0.59,0.14)
CPI	Calculus deposits vs. Healthy gum /No bleeding or calculus	‑0.26 (‑0.68,0.15)	‑0.16 (‑0.53,0.24)
	Bleeding on probing vs. Healthy gum /No bleeding or calculus	‑0.02 (‑0.50,0.47)	‑0.03 (‑0.47,0.41)
	Calculus deposits vs. Bleeding on probing	‑0.24 (‑0.61,0.13)	‑0.13 (‑0.46,0.21)
DMFT		0.08 (‑0.08,0.25)	0.09 (‑0.07,0.24)

Data in boldface: 95% credible interval excluded 0, considered as statistically significant at 0.05 level of significance

**Table 5:** Longitudinal explanatory GRM (Model 2): Regression coefficients with 95% credible intervals

Factors	Change	CPQ_11‑14_ RSF:8	CPQ_11‑14_ ISF:8
Daily brushing frequency	(1) Brush less than twice ➔More than twice vs. No change	0.33 (‑0.20,0.87)	0.06 (‑0.50,0.58)
	(2) Brush more than twice ➔More than twice vs. No change	‑0.02 (‑0.50,0.46)	0.00 (‑0.48,0.49)
	Contrast: (2) vs. (1)	‑0.36 (‑1.04,0.34)	‑0.06 (‑0.72,0.64)
Snacking frequency between meals	Less snack intake between meals vs. No change	‑0.13 (‑0.46,0.20)	‑0.15 (‑0.48,0.16)
	More snack intake between meals vs. No change	0.12 (‑0.21,0.46)	0.01 (‑0.33,0.34)
	More vs. Less snack intake between meals	0.25 (‑0.13,0.64)	0.17 (‑0.21,0.54)
Parents employment status	(1) Become both parents employed vs. No change	0.09 (‑0.43,0.61)	0.31 (‑0.18,0.80)
	(2) At least one parent become unemployed vs. No change	‑0.09 (‑0.64,0.44)	‑0.24 (‑0.81,0.31)
	Contrast: (2)‑(1)	‑0.19 (‑0.91,0.54)	‑0.55 (‑1.26,0.13)
Monthly family income	Increased family income vs. No change	0.02 (‑0.28,0.33)	‑0.16 (‑0.47,0.14)
	Decreased family income vs. No change	**0.50 (0.07,0.96)**	0.36 (‑0.08,0.81)
	Decreased vs. Increased income	**0.48 (0.02,0.96)**	**0.52 (0.05,1.00)**
CPI	Improved CPI vs. No change	‑0.26 (‑0.76,0.25)	‑0.03 (‑0.54,0.46)
	Worsened CPI vs. No change	0.17 (‑0.21,0.54)	0.04 (‑0.33,0.40)
	Worsened vs. Improved CPI	0.42 (‑0.15,1.03)	0.06 (‑0.50,0.66)
DMFT	Increased DMFT	‑0.03 (‑0.10,0.05)	‑0.02 (‑0.09,0.05)

Data in boldface: 95% credible interval excluded 0, considered as statistically significant at 0.05 level of significance

**Table 6:** Longitudinal explanatory GRM (Model 3): Regression coefficients with 95% credible intervals

Factors	Comparison of regression coefficients	Baseline		Follow-up	
		CPQ_11‑14_ RSF:8	CPQ_11‑14_ ISF:8	CPQ_11‑14_ RSF:8	CPQ_11‑14_ ISF:8
Gender	Boys vs. Girls	‑0.16 (‑0.40,0.07)	‑0.06 (‑0.33,0.20)	‑0.28 (‑0.57,0.03)	‑0.26 (‑0.57,0.05)
Both parents employed	Yes vs. No	**‑0.23 (‑0.47,0.00)**	**‑0.36 (‑0.63,‑0.11)**	0.12 (‑0.23,0.44)	0.23 (‑0.07,0.52)
Monthly family income	>$40,000 vs. <$10,000	0.08 (‑0.37,0.51)	0.17 (‑0.30,0.65)	‑0.01 (‑0.59,0.55)	0.08 (‑0.49,0.68)
	$30,000‑$40,000 vs. <$10,000	0.33 (‑0.11,0.77)	0.25 (‑0.23,0.73)	‑0.04 (‑0.54,0.46)	0.32 (‑0.17,0.83)
	$20,000‑$30,000 vs. <$10,000	0.19 (‑0.14,0.57)	0.22 (‑0.15,0.61)	0.17 (‑0.28,0.64)	0.21 (‑0.24,0.67)
	$10,000‑$20,000 vs. <$10,000	0.13 (‑0.15,0.42)	0.21 (‑0.12,0.53)	‑0.02 (‑0.57,0.52)	0.24 (‑0.30,0.78)
	>$40,000 vs. $30,000‑$40,000	‑0.25 (‑0.70,0.18)	‑0.08 (‑0.57,0.40)	0.19 (‑0.32,0.69)	0.13 (‑0.34,0.60)
	>$40,000 vs. $20,000‑$30,000	‑0.12 (‑0.53,0.30)	‑0.05 (‑0.52,0.38)	0.22 (‑0.19,0.61)	‑0.11 (‑0.50,0.27)
	>$40,000 vs. $10,000‑$20,000	‑0.06 (‑0.47,0.34)	‑0.05 (‑0.47,0.39)	‑0.29 (‑0.86,0.27)	‑0.03 (‑0.61,0.59)
	$30,000‑$40,000 vs. $20,000‑$30,000	0.14 (‑0.28,0.55)	0.03 (‑0.44,0.48)	‑0.28 (‑0.83,0.27)	‑0.11 (‑0.69,0.46)
	$30,000‑$40,000 vs. $10,000‑$20,000	0.20 (‑0.19,0.58)	0.04 (‑0.40,0.46)	‑0.26 (‑0.74,0.26)	‑0.35 (‑0.86,0.18)
	$20,000‑$30,000 vs. $10,000‑$20,000	0.06 (‑0.25,0.38)	0.01 (‑0.32,0.37)	**‑0.48 (‑0.92,‑0.01)**	‑0.24 (‑0.67,0.21)
Father’s education	College graduate or above vs. Below secondary school	‑0.43 (‑0.95,0.07)	‑0.29 (‑0.90,0.30)	0.13 (‑0.60,0.77)	0.23 (‑0.50,0.99)
	Senior secondary school (S4‑7) vs. Below secondary school	**‑0.73 (‑1.12,‑0.38)**	**‑0.67 (‑1.15,‑0.25)**	‑0.13 (‑0.67,0.30)	‑0.21 (‑0.70,0.35)
	Junior Secondary school (S1‑3) vs. Below secondary school	**‑0.71 (‑1.09,‑0.36)**	**‑0.69 (‑1.11,‑0.27)**	0.00 (‑0.50,0.45)	‑0.13 (‑0.61,0.37)
	College graduate or above vs. Senior secondary school (S4‑7)	0.30 (‑0.11,0.69)	0.38 (‑0.07,0.83)	0.25 (‑0.26,0.79)	0.42 (‑0.10,0.98)
	College graduate or above vs. Junior secondary school (S1‑3)	0.28 (‑0.20,0.76)	0.40 (‑0.12,0.92)	0.11 (‑0.52,0.78)	0.36 (‑0.30,1.01)
	Senior secondary school (S4‑7) vs. Junior secondary school (S1‑3)	‑0.02 (‑0.33,0.29)	0.02 (‑0.31,0.35)	‑0.13 (‑0.57,0.27)	‑0.08 (‑0.47,0.34)
Mother’s education	College graduate or above vs. Below secondary school	0.30 (‑0.19,0.80)	0.31 (‑0.26,0.83)	‑0.26 (‑0.89,0.37)	‑0.30 (‑0.95,0.27)
	Senior secondary school (S4‑7) vs. Below secondary school	0.24 (‑0.14,0.61)	0.41 (‑0.01,0.83)	‑0.29 (‑0.74,0.20)	‑0.11 (‑0.60,0.38)
	Junior Secondary school (S1‑3) vs. Below secondary school	**0.43 (0.03,0.83)**	**0.48 (0.02,0.91)**	‑0.20 (‑0.69,0.30)	‑0.06 (‑0.57,0.43)
	College graduate or above vs. Senior secondary school (S4‑7)	0.06 (‑0.30,0.44)	‑0.11 (‑0.54,0.29)	0.02 (‑0.43,0.53)	‑0.20 (‑0.67,0.26)
	College graduate or above vs. Junior secondary school (S1‑3)	‑0.13 (‑0.55,0.29)	‑0.18 (‑0.62,0.26)	‑0.06 (‑0.61,0.50)	‑0.25 (‑0.78,0.27)
	Senior secondary school (S4‑7) vs. Junior secondary school (S1‑3)	‑0.19 (‑0.49,0.10)	‑0.07 (‑0.38,0.26)	‑0.09 (‑0.48,0.28)	‑0.05 (‑0.44,0.35)
Brushing frequency	Twice or more daily vs. Less than twice daily	**‑0.29 (‑0.54,‑0.05)**	**‑0.44 (‑0.71,‑0.16)**	‑0.13 (‑0.44,0.20)	‑0.19 (‑0.49,0.14)
Snacking frequency between meals	3 times or more vs. None	**0.64 (0.23,1.07)**	**0.64 (0.17,1.11)**	‑0.29 (‑0.85,0.25)	‑0.24 (‑0.76,0.32)
	Twice vs. None	**0.33 (0.01,0.65)**	0.21 (‑0.16,0.57)	0.21 (‑0.21,0.65)	0.21 (‑0.20,0.65)
	Once vs. None	‑0.01 (‑0.27,0.25)	‑0.03 (‑0.34,0.26)	0.09 (‑0.29,0.41)	0.16 (‑0.20,0.54)
	3 times or more vs. Twice	0.32 (‑0.13,0.74)	0.43 (‑0.05,0.92)	‑0.51 (‑1.08,0.04)	‑0.45 (‑1.00,0.09)
	3 times or more vs. Once	**0.66 (0.26,1.05)**	**0.67 (0.24,1.11)**	‑0.38 (‑0.89,0.13)	‑0.39 (‑0.88,0.10)
	Twice vs. Once	**0.34 (0.07,0.62)**	0.24 (‑0.06,0.54)	0.13 (‑0.22,0.51)	0.06 (‑0.29,0.40)
CPI	Calculus deposits vs. Healthy gum /No bleeding or calculus	0.21 (‑0.06,0.47)	0.26 (‑0.11,0.59)	0.08 (‑0.39,0.48)	0.17 (‑0.27,0.62)
	Bleeding on probing vs. Healthy gum /No bleeding or calculus	**0.33 (0.02,0.66)**	0.34 (‑0.07,0.72)	‑0.29 (‑0.88,0.27)	0.03 (‑0.51,0.59)
	Calculus deposits vs. Bleeding on probing	‑0.12 (‑0.39,0.15)	‑0.08 (‑0.36,0.21)	0.37 (‑0.06,0.79)	0.14 (‑0.29,0.56)
DMFT		‑0.04 (‑0.16,0.07)	‑0.11 (‑0.25,0.02)	0.00 (‑0.06,0.06)	0.00 (‑0.06,0.05)

Data in boldface: 95% credible interval excluded 0, considered as statistically significant at 0.05 level of significance

Again, WinBUGS was used to estimate the credible intervals (Additional file 1). Association of explanatory variables with OHRQoL at respective occasions were established when 95% credible intervals excluded 0 or differences in any pair of γ’s within a factor excluded 0.

To rule out the change in OHRQoL score due to change in interpretation of the items over 3 years, discriminatory and threshold parameters of items that were not invariant were allowed to vary across baseline and follow-up.

## Results

### Descriptive statistics

Six hundred sixty eight sets of parental and student questionnaires from 12 years old children were collected; 436 (65.3%) students were followed up successfully after 3 years. At baseline, 19 cases with more than 2 missing items in CPQ were excluded; 649 were used in the analysis. In the 3-year follow-up, 415 cases were used in the analysis (after excluding 21 cases with more than 2 missing items in CPQ). The mean scores for CPQ_11–14_ RSF:8 and ISF:8 at baseline were respectively 7.0 (SD = 3.8, range = 0–20) and 7.4 (SD = 3.6, range = 0–21). The mean scores of CPQ_11–14_ RSF:8 and ISF:8 at follow-up were respectively 6.2 (SD = 3.7, range = 0–17) and 7.2 (SD = 3.8, range = 0–19). The median class of monthly family income was $10,000–$20,000 at both baseline and follow-up. Regarding the oral health behaviors, more than a quarter of the sample brushed teeth less than twice a day and only less than a quarter did not eat snack between meals. For the oral health status, only about one in ten had healthy gum while majority of them bled on probing or had calculus. The mean DMFT on baseline was 0.55 while that in the follow-up had increased to 1.70. The frequency distributions of other factors considered at both baseline and follow-up are shown in Table 1.

### Explanatory GRM

Table 2 shows the estimated median and the 95% credible intervals of the regression coefficients and the difference of each pair of levels within each factor for 12 years old children. Respondents with more frequent tooth brushing, less snacks consumption between meals, and with both parents employed showed a better OHRQoL. However, people with worse gum status were found having a worse OHRQoL. Regarding parental education, it is noted that significantly better OHRQoL was found for those with fathers attained secondary compared to below secondary education while significantly worse OHRQoL for those with mothers attained secondary compared to below secondary education. No significant differences were found among other comparisons for father’s or mother’s education.

### GRM for assessing longitudinal invariance with external anchor items

A longitudinal GRM model allowing parameter drift was fitted with the 5 global rating anchor items. Results on longitudinal invariance are shown in Table 3. No significant change in discriminatory parameters of RSF:8 or ISF:8 was found. This indicated the absence of non-uniform longitudinal invariance. Two items form CPQ_11–14_ RSF:8 and 2 items from ISF:8 had significant threshold parameters drift. In CPQ_11–14_ RSF:8, the items were “Argued with other children or your family” and “Teased/ called names by other children”. In CPQ_11–14_ ISF:8, these items were “Difficult to drink or eat hot or cold foods” and “Irritable/ frustrated”. For the item “Argued with other children or your family”, children at follow-up were prone to choose “Never” = 0 compared to baseline even their OHRQoL levels have not changed. For the item “Teased/ called names by other children”, children at follow-up were more likely to response to “Never” = 0 or “Once/ twice” = 1. For the item “Difficult to drink or eat hot or cold foods”, children at follow-up were prone to choose “Once/ twice” = 1 or higher response options. For the item “Irritable/ frustrated”, children at follow-up were more likely to choose “Sometimes” = 2, or higher response options. Of the 4 items with longitudinal invariance, 3 items came from the domains of emotional well-being and social well-being while the other one came from the domain of functional limitations.

It was found that the OHRQoL was significantly improved over 3 years with change in the mean latent score = − 0.29 and the 95% credible interval (− 0.44, − 0.15) excluded 0. The standard deviation of OHRQoL in 3-year follow up has increased from 1.01 to 1.35. Significant positive correlation was also found between baseline and follow-up latent OHRQoL score (*r* = 0.43) with its credible interval (0.30, 0.58) that excluded 0.

### Longitudinal explanatory GRM

Tables 4, 5and 6 present the results of the longitudinal models with adjustment for longitudinal invariance by allowing the threshold parameter of the 4 items (with significant parameter drift) to vary. In Model 1, baseline explanatory variables were used to predict the change over 3 years (Table 4). Respondents who consumed more snacks at baseline had significantly more improvement in OHRQoL over 3 years. Table 5 presents the results of a similar longitudinal model. Instead of baseline predictors, the change in demographics variables and the change in oral health behavior/status over the 3 years were used to predict the change in OHRQoL (Model 2). For participants with decreased income, their OHRQoL had worsened over 3 years. Table 6 presents the results of Model 3 that used 12 years old and 15 years old conditions to explain baseline and follow-up OHRQoL respectively, while adjusting for longitudinal invariance. Variables significantly associated with 12-years old OHRQoL were similar to that in cross-sectional analysis. More frequent tooth brushing, a lower mother education level (lower secondary vs. primary) and a higher father education level (lower secondary vs. primary, upper secondary vs. primary) was associated with better OHRQoL. Respondents with both parents employed, less snacks consumption between meals and better gum conditions can predict 12 years old OHRQoL. In 15 years old, no variables were significantly associated with OHRQoL.

## Discussions

### Longitudinal invariance

Three of the four longitudinally biased items were from the domains of emotional and social well-being, namely “Argue with other children or family”, “Teased/ called names by other children” and “Irritable/frustrated”. In short, children tended to choose a lower response options in items concerning emotional and social well-being in the follow-up study. One way to adjust longitudinal invariance is to remove the problematic item. This may however result in an instrument with questionable content validity. Allowing invariant parameters to be varied provides an alternative adjustment method for longitudinal invariance while preserving the content validity.

The investigation of longitudinal invariance also highlighted the importance of testing longitudinal invariance especially when items concern emotions or perceptions which possibly change over time. Moreover, adjustment for longitudinal invariance also enables the questionnaire to be appropriate even if the participants’ age was one year exceeded the CPQ’s target age range of 11–14 years.

### Oral health status and OHRQoL

Dental caries experience did not significantly associate with OHRQoL in the present study. This was consistent only to the findings from few countries. Table 7 shows studies concerning dental caries status and OHRQoL in adolescents in different countries. It reveals that most studies concluded the significant association had a higher mean DMFT or prevalence compared to that in present study. Studies found no such significant association had a comparable mean DMFT or prevalence to that in the present study, for instance, a study in Dunedin and a previous study in Hong Kong. Although the hypothesis “*Dental caries predict OHRQoL only in higher impact populations.*” requires a meta-analysis to conclude, the studies in Table 7 and the present study suggest that a higher disease level may be required for detecting the impact of dental caries on OHRQoL because mild dental caries, even untreated, may not be painful and hardly affect the person. Other studies that found the significant association between oral health status and OHRQoL are often in large sample or diseased group [21].

**Table 7:** Comparison of dental caries level in different geographic population

Age range	Population	Dental caries level	Study
Non‑Significant relationship to OHRQoL			
12, 15,18	Hong Kong	Mean DMFT at age 12,15 and 18 = 0.6, 1.1, 1.5 respectively	[29]
12‑13	Dunedin, New Zealand	Mean DMFS = 1.6	[26]
		DMFS> 0 = 44.2%	
Significant relationship to OHRQoL			
14‑17	Santa Catarina State, Brazil	Mean DMFT = 5.4	[30]
		DMFT> 0 = 88.2%	
11‑13	Iran	Mean DMFT = 1.8	[31]
12 & 15	Modinagar, India	DMFT> 0 = 63%	[32]
12 & 15	Thailand	Mean DMFT =1.3‑2.1 (12 years old), Mean DMFT = 1.9‑2.9 (15 years old) in different regions	[33]
15‑19	Brazil general population	> 0 untreated caries = 46.1%	[34]
10‑14	Thailand	Mean DMFT = 1.97 (Association found only in 6 month follow‑up)	[21]

### SES and OHRQoL

Many studies have attempted to relate the social economic status to OHRQoL but mixed results have been found. Inconsistent results may be attributed to whether the analysis has been adjusted to confounding factors. Also, different studies have employed different variables as a proxy of SES: parents’ education levels, occupations, parents’ age, parents’ attitude, family income, type of flat, household crowding, number of siblings and so on [22]. In this study, the parents’ education level, family income and whether both parents are working were used to measure the SES. There were significant differences in OHRQoL across some subgroups of parents’ education level. But no clear indication of associations between OHRQoL and higher/ lower education levels was observed.

Socioeconomic status often affects OHRQoL through the limited access of dental utilities due to material deprivation [23]. It is unlikely to be the case in Hong Kong because government’s SDCS provides children from primary school (equivalent to US grade 1 to 6) an annual dental checkup. In our sample, 98% of the school children participated in the scheme or had private dental insurance. Almost all children have received adequate dental care regardless of their family income. This may explain the weak association between income and OHRQoL in Hong Kong. However, SDCS was no longer provided for adolescents after 12 years old. Adolescents with increased family income over the age of 12–15 had only little OHRQoL improvement over these 3 years. This concurs with the hypothesis that the lower economic status leads to inadequate access of dental utilities, and thus OHRQoL. This suggests that a specific scheme taking care of children from low income family is needed rather than general measures like extending SDCS to older ages.

### Weakened associations at 15 years old follow-up

All predictors found predictive in OHRQoL of 12 years old adolescents (oral health behaviors, gum status, some socioeconomic variables) became insignificant in the 15 years old follow-up with adjustment for longitudinal invariance. This may be related to the improvement of the OHRQoL over 3 years. When an already low-impact population has further improved, the OHRQoL becomes better in a homogeneous manner. The baseline characteristics (shown in Table 1) of those children lost to follow up were compared with the rest of respondents. No significant difference were found in all variables, except for daily brushing frequencies (Twice or more: 75.9% in follow-up vs. 65.0% in lost follow-up), CPI (Calculus deposit: 62.2% in follow-up vs. 69.7% in lost follow-up) and DMFT (Mean DMFT: 0.47 in follow-up vs. 0.71 in lost follow-up). It makes detecting effect of factors even harder, subsequently resulting in a failure to identify factor significantly associated to OHRQoL of adolescent in 15-years old.

The weakened association at 15 years old may due to the “regress to the mean” which describes the trend that significant associations tended to become insignificant when the variables are measured in the second time because of the random error [24]. Respondents who consumed more snacks had significantly greater improvement in OHRQoL over 3 years and this appear to be counter-intuitive. This is suspicious to the “regress to the mean” effect especially the snacking frequency is highly susceptible to random error due to the difficulty to recall exactly the snack intake and neglecting the amount and types of snack consumed.

### Limitation

Relying on anchor to establish item longitudinal invariance is sometimes criticized because invariance properties of the anchor itself is doubted. This study address this concern by choosing relatively large number of global self-rating items reflecting their own perception about OHRQoL and its impact on daily life.

Many studies suggested that psychological characteristics played an important role in coping with unfavorable conditions, for instance, poor dental aesthetics [25]. No data about sense of coherence, self-esteem and confidence were collected, which have been proven to be potential predictors of OHRQoL [22, 26, 27]. Moreover, malocclusion status directly related to facial appearance and thus may affect OHRQoL directly. Association of OHRQoL and malocclusion status has been shown in both general population and studies confined to disease specific groups [28]. Unfortunately, malocclusion data was not available to be used in this study.

Being part of a larger scale cohort study “Children of 1997”, it is a representative sample of the general Hong Kong adolescents. It should be noted that this is a study on a low impact population of dental caries and OHRQoL. Also, the lost to follow-up respondents had slightly worse oral health status, this dataset may fail to capture their OHRQoL development which may drive more actionable insight for oral health policies. When generalizing the results, one should aware of the cultural difference and the relevant local dental policies which vary across countries.

## Conclusion

In conclusion, IRT model is extended to fit longitudinal data and incorporate explanatory variables of OHRQoL. This study also illustrated the use of IRT in detection and adjustment of longitudinal invariance. The OHRQoL at 12 was associated with demographic background variables, oral health behavior and status; however, these associations were not observed in 15-years old children after adjustment for longitudinal invariance. For children with decreased income, they have worsened OHRQoL. The results provide important information to oral healthcare researchers and policymakers.

## Additional file

Additional file 1: WINBUGS Syntax. (DOCX 16 kb)

## Abbreviations

CPI: Community Periodontal Index
CPQ_11–14_: Child Perception Questionnaires
DMFT: Decayed missing filled teeth
GRM: Graded response model
ICC: Intra-class correlation
IRT: Item response theory
ISF:8: 8-item Short form CPQ_11–14_ obtained by item impact method
MCMC: Monte Carlo Markov Chain
OHRQoL: Oral health-related quality of life
RSF:8: 8-item Short form CPQ_11–14_ obtained by regression method
SD: Standard deviation
SDCD: School Dental Care Service
SES: Socio economic status

## References

[1] Wirth R, Edwards MC (2007). Item factor analysis: current approaches and future directions. Psychol Methods.

[2] Yau DT, Wong MC, Lam KF, McGrath C (2015). Evaluation of psychometric properties and differential item functioning of 8-item child perceptions questionnaires using item response theory. BMC Public Health.

[3] Wong HM, McGrath CP, King NM (2011). Rasch validation of the early childhood oral health impact scale. Community Dent Oral Epidemiol.

[4] Gilchrist F, Rodd H, Deery C, Marshman Z (2014). Assessment of the quality of measures of child oral health-related quality of life. BMC Oral Health.

[5] McGrath C, Pang HN, Lo E, King NM, HÄGG U, Samman N (2008). Translation and evaluation of a Chinese version of the child oral health-related quality of life measure. Int J Paediatr Dent.

[6] Wong MC, Lau AW, Lam KF, McGrath C, Lu HX (2011). Assessing consistency in oral health-related quality of life (OHRQoL) across gender and stability of OHRQoL over time for adolescents using structural equation modeling. Community Dent Oral Epidemiol.

[7] Hetherington EM, Parke RD, Locke VO (1999). Child psychology: a contemporary viewpoint.

[8] Meade AW, Lautenschlager GJ, Hecht JE (2005). Establishing measurement equivalence and invariance in longitudinal data with item response theory. Int J Testing.

[9] Golembiewski RT, Billingsley K, Yeager S (1976). Measuring change and persistence in human affairs: types of change generated by OD designs. J Applied Behavioral Science.

[10] Pastor DA (2003). The use of multilevel item response theory modeling in applied research: an illustration. Appl Meas Educ.

[11] Adams RJ, Wilson M, Wu M (1997). Multilevel item response models: an approach to errors in variables regression. J Educational Behavioral Statistics.

[12] Briggs DC (2008). Using explanatory item response models to analyze group differences in science achievement. Appl Meas Educ.

[13] Gorter R, Fox J-P, Apeldoorn A, Twisk J (2016). Measurement model choice influenced randomized controlled trial results. J Clin Epidemiol.

[14] Peng S, Wong H, King N, McGrath C (2014). Association between dental caries and adiposity status (general, central, and peripheral adiposity) in 12-year-old children. Caries Res.

[15] Jokovic A, Locker D, Stephens M, Kenny D, Tompson B, Guyatt G (2002). Validity and reliability of a questionnaire for measuring child oral-health-related quality of life. J Dent Res.

[16] WHO (2013). Oral health surveys: basic methods.

[17] Wilson M, De Boeck P, De Boeck P, Wilson M (2004). Descriptive and explanatory item response models. Explanatory item response models: a genralized linear and nonlinear approach.

[18] Spiegelhalter D, Thomas A, Best N, Lunn D (2003). WinBUGS user manual. Version 1.4.

[19] Tanner MA (1991). Tools for statistical inference: observed data and data augmentation methods.

[20] Kolen MJ (1984). Effectiveness of analytic smoothing in equipercentile equating. J Educational Behavioral Statistics..

[21] Gururatana O, Baker SR, Robinson PG (2014). Determinants of children’s oral-health-related quality of life over time. Community Dent Oral Epidemiol.

[22] Kumar S, Kroon J, Lalloo R (2014). A systematic review of the impact of parental socio-economic status and home environment characteristics on children’s oral health related quality of life. Health Qual Life Outcomes.

[23] Paula JS, Leite I, Almeida AB, Ambrosano G, Pereira AC, Mialhe FL (2012). The influence of oral health conditions, socioeconomic status and home environment factors on schoolchildren’s self-perception of quality of life. Health Qual Life Outcomes.

[24] Barnett AG, van der Pols JC, Dobson AJ (2005). Regression to the mean: what it is and how to deal with it. Int J Epidemiol.

[25] Inglehart MR, Bagramian RA (2002). Oral health-related quality of life.

[26] Foster Page LA, Thomson WM, Ukra A, Farella M (2013). Factors influencing adolescents’ oral health-related quality of life (OHRQoL). Int J Paediatr Dent.

[27] Foster Page LA, Thomson WM, Ukra A, Baker SR (2013). Clinical status in adolescents: is its impact on oral health-related quality of life influenced by psychological characteristics?. Eur J Oral Sci.

[28] Liu Z, McGrath C, Hägg U (2009). The impact of malocclusion/orthodontic treatment need on the quality of life: a systematic review. Angle Orthod.

[29] Lu H, Wong M, Lo E, McGrath C (2011). Trends in oral health from childhood to early adulthood: a life course approach. Community Dent Oral Epidemiol.

[30] Biazevic MGH, Rissotto RR, Michel-Crosato E, Mendes LA, Mendes MOA (2008). Relationship between oral health and its impact on quality of life among adolescents. Brazilian Oral Res.

[31] Bakhtiar M, Mohammadi TM, Hajizamani A, Vossoughi M (2014). Association of oral health indicators with quality-of-life related to oral health among Iranian adolescent. J Int Oral Health.

[32] Basavaraj P, Sunil M, Nagarajappa R, Ashish S, Ramesh G (2014). Correlation between oral health and child-OIDP index in 12-and 15-year-old children from Modinagar. India Asia-Pacific J Public Health.

[33] Krisdapong S, Prasertsom P, Rattanarangsima K, Sheiham A (2013). Sociodemographic differences in oral health-related quality of life related to dental caries in thai school children. Community Dent Health.

[34] Peres KG, Cascaes AM, Leão ATT, Côrtes MI, Vettore MV (2013). Sociodemographic and clinical aspects of quality of life related to oral health in adolescents. Revista de Saude Publica.

