# Supplementary material for: Longitudinal measurement invariance and explanatory IRT models for adolescents’ oral health-related quality of life
Source: Health Qual Life Outcomes. 2018 Apr 11;16:60. doi: 10.1186/s12955-018-0879-x (PMC5896083; doi:10.1186/s12955-018-0879-x)
Supplement: Supplementary file 1 — WINBUGS Syntax. (DOCX 16 kb) [file 12955_2018_879_MOESM1_ESM.docx]

**WINBUGS Syntax:**

**Baseline model:**

model{

for (i in 1:n){

for (j in 3:p){

y[i,j]~dcat(prob[i,j‑2,1:5])}

e[i]~dnorm(0,tau.e)}

for (i in 1:n) {

for (j in 3:p){

for (k in 1:4){

logit(P[i,j‑2,k])<‑kappa[j‑2,k]‑alpha[j‑2]*theta[i]}

P[i,j‑2,5]<‑1}}

for (i in 1:n) {

for (j in 3:p){

prob[i,j‑2,1]<‑P[i,j‑2,1]

for (k in 2:5){

prob[i,j‑2,k]<‑P[i,j‑2,k]‑P[i,j‑2,k‑1]}}}

for (j in 3:p){

for (k in 1:4){

beta[j‑2,k]<‑kappa[j‑2,k]*pow(alpha[j‑2],‑1)}}

for (i in 1:n){

# Some explanatory variables omitted

theta[i]<‑gam[1]*gender[i]+gam[2]*Brush[i]+…+gam[34]*DMFT[i]+e[i] }

p.alpha[1]<‑1

for (j in 3:p‑1) {

alpha[j‑2]~dgamma(0.001,0.001)

p.alpha[j‑2+1]<‑p.alpha[j‑2]*alpha[j‑2]}

alpha[p‑2]<‑pow(p.alpha[p‑2],‑1)

for (i in 1:35){

gam[i]~dnorm(0,0.001)}

tau.e<‑pow(sigma,‑1)

sigma~dgamma(0.001,0.001)

for (j in 3:p) {

for (k in 1:4){

kappa.star[j‑2,k]~dnorm(m.kappa,pr.kappa)

kappa[j‑2,k]<‑ranked(kappa.star[j‑2,1:4],k)}}

pr.kappa<‑pow(s.kappa,‑2)

s.kappa~dnorm(0,0.0001)

m.kappa~dnorm(0,0.0001)}:

**Model 1 (baseline covariates) & Model 2 (change between baseline and follow-up as covariates):**

model{

for (t in 1:2){

for (i in 1:n){

for (j in 1:p){

y[i,j,t]~dcat(prob[i,j,t,1:4])}}}

for (t in 1:1){

for (i in 1:n) {

for (j in 1:p){

for (k in 1:3){

logit(P[i,j,k,t])<‑kappa[j,k,t]‑alpha[j]*theta[i] }

P[i,j,4,t]<‑1 }}}

for (t in 2:2){

for (i in 1:n) {

for (j in 1:p){

for (k in 1:3){

logit(P[i,j,k,t])<‑kappa[j,k,t]‑alpha[j]*(theta[i]+delta[i]) }

P[i,j,4,t]<‑1 }}}

for (t in 1:2){

for (i in 1:n) {

for (j in 1:p){

prob[i,j,t,1]<‑P[i,j,1,t]

for (k in 2:4){

prob[i,j,t,k]<‑P[i,j,k,t]‑P[i,j,k‑1,t]}}}}

for (t in 1:2){

for (j in 1:p){

for (k in 1:3){

beta[j,k,t]<‑kappa[j,k,t]*pow(alpha[j],‑1)}}}

for (i in 1:n){

# Some explanatory variables omitted

delta[i]<‑gam[1]*gender[i]+gam[2]*Brush12[i]+…+gam[34]*DMFT12[i]+e[i]

e[i]~dnorm(m.e,pr.e)

theta[i]~dnorm(0,pr.theta)}

p.alpha[1]<‑1

for (j in 1:p‑1) {

alpha[j]~dgamma(0.1,0.1)

p.alpha[j+1]<‑p.alpha[j]*alpha[j]}

alpha[p]<‑pow(p.alpha[p],‑1)

for (t in 1:1){

for (j in 1:p) {

for (k in 1:3){

kappa.star[j,k,t]~dnorm(0,0.001)

kappa[j,k,t]<‑ranked(kappa.star[j,1:3,t],k)}}}

#allow item parameters of items 4 & 5 to be vary

for (t in 2:2){

for (j in 4:5) {

for (k in 1:3){

kappa.star[j,k,t]~dnorm(0,0.001)

kappa[j,k,t]<‑ranked(kappa.star[j,1:3,t],k)}}}

for (j in 1:3) {

for (k in 1:3){

kappa[j,k,2]<‑kappa[j,k,1]}}

for (j in 6:8) {

for (k in 1:3){

kappa[j,k,2]<‑kappa[j,k,1]}}

m.e~dnorm(0,0.0001)

pr.e<‑pow(s.e,‑2)

s.e~dgamma(0.01,0.01)

pr.theta<‑pow(s.theta,‑2)

s.theta~dgamma(0.01,0.01)}

**Model 3:**

model{

for (t in 1:2){

for (i in 1:n){

for (j in 1:p){

y[i,j,t]~dcat(prob[i,j,t,1:4])}}}

for (t in 1:2){

for (i in 1:n) {

for (j in 1:p){

for (k in 1:3){

logit(P[i,j,k,t])<‑kappa[j,k,t]‑alpha[j]*theta[i,t]}

P[i,j,4,t]<‑1 }}}

for (t in 1:2){

for (i in 1:n) {

for (j in 1:p){

prob[i,j,t,1]<‑P[i,j,1,t]

for (k in 2:4){

prob[i,j,t,k]<‑P[i,j,k,t]‑P[i,j,k‑1,t]}}}}

for (t in 1:2){

for (j in 1:p){

for (k in 1:3){

beta[j,k,t]<‑kappa[j,k,t]*pow(alpha[j],‑1)}}}

for (i in 1:n){

e[i,1:2]~dmnorm(m.e[], pr.e[,])

# Some explanatory variables omitted

theta[i,1]<‑gam[1]*gender[i]+gam[2]*Brush12[i]…+gam[34]*DMFT12[i]+e[i,1]

theta[i,2]<‑del[1]*gender[i]+del[2]*Brush15[i]+…+del[34]*DMFT15[i]+e[i,2]}

m.e[1]<‑0

m.e[2]~dnorm(0,0.001)

L.e[1,1]~ dgamma(0.001,0.001)

L.e[2,2]~ dgamma(0.001,0.001)

L.e[2,1]~dnorm(0,0.001)

L.e[1,2]<‑0

for (i in 1:2){

for (j in 1:2){

sigma.e[i,j]<‑inprod(L.e[i, 1:2],L.e[j, 1:2]) }}

pr.e[1:2,1:2]<‑inverse(sigma.e[,])

p.alpha[1]<‑1

for (j in 1:p‑1) {

alpha[j]~dgamma(0.001,0.001)

p.alpha[j+1]<‑p.alpha[j]*alpha[j]}

alpha[p]<‑pow(p.alpha[p],‑1)

for (t in 1:1){

for (j in 1:p) {

for (k in 1:3){

kappa.star[j,k,t]~dnorm(m.kappa,pr.kappa)

kappa[j,k,t]<‑ranked(kappa.star[j,1:3,t],k)}}}

for (t in 2:2){

for (j in 4:5) {

for (k in 1:3){

kappa.star[j,k,t]~dnorm(0,0.001)

kappa[j,k,t]<‑ranked(kappa.star[j,1:3,t],k)}}}

for (j in 1:3) {

for (k in 1:3){

kappa[j,k,2]<‑kappa[j,k,1]}}

for (j in 6:8) {

for (k in 1:3){

kappa[j,k,2]<‑kappa[j,k,1]}}

pr.kappa<‑pow(s.kappa,‑2)

for (i in 1:34){

gam[i]~dnorm(0,0.001)}

for (i in 1:34){

del[i]~dnorm(0,0.001)}}
